# Fibroblast-growth factor 23 promotes terminal differentiation of ATDC5 cells

**DOI:** 10.1371/journal.pone.0174969

**Published:** 2017-04-13

**Authors:** Mathilde Guibert, Adeline Gasser, Hervé Kempf, Arnaud Bianchi

**Affiliations:** UMR 7365 CNRS-Université de Lorraine « Ingénierie Moléculaire et Physiopathologie Articulaire » (IMoPA), Biopôle de l’Université de Lorraine, Campus Biologie-Santé, Vandœuvre-lès-Nancy, France; Nihon University School of Medicine, JAPAN

## Abstract

**Objectives:**

Fibroblast Growth Factor 23 (FGF23) is well documented as a crucial player in the systemic regulation of phosphate homeostasis. Moreover, loss-of-function experiments have revealed that FGF23 also has a phosphate-independent and local impact on skeletogenesis. Here, we used ATDC5 cell line to investigate the expression of FGF23 and the role it may play locally during the differentiation of these cells.

**Methods:**

ATDC5 cells were differentiated in the presence of insulin, and treated with recombinant FGF23 (rFGF23), inorganic phosphate (Pi) and/or PD173074, an inhibitor of FGF receptors (FGFRs). The mRNA expressions of *FGF23*, *FGFRs* and markers of hypertophy, *Col X and MMP13*, were determined by qPCR analysis and FGF23 production was assessed by ELISA. FGFR activation was determined by immunoprecipitation and immunoblotting.

**Results:**

FGF23 mRNA expression and production were increased during ATDC5 differentiation. At D28 in particular, rFGF23 stimulation increased hypertrophic markers expression, as *Col X* and *MMP13*, and mineralization. A synergic effect of Pi and rFGF23 stimulation was observed on these markers and on the mineralization process. The use of PD173074, a pan-FGFR inhibitor, decreased terminal differentiation of ATDC5 by preventing rFGF23 pro-hypertrophic effects.

**Conclusions:**

Altogether, our results provide evidence that FGF23 plays an important role during differentiation of ATDC5 cell line, by promoting both hypertrophy and mineralization.

## Introduction

Chondrogenesis is a tightly regulated process that results in the formation of the cartilage anlage and leads to endochondral ossification during skeletal development. Tremendous work over the past decades has revealed the role of various signaling molecules and their crosstalk in the orderly process of endochondral ossification that takes place within the growth plate. BMP, Wnt, hedgehog, PTH or FGF signaling pathways play sequential or concomittant roles in the various steps of chondrogenesis, including mesenchymal condensation, chondrocyte proliferation and chondrocyte hypertrophy ultimately leading to the calcification of the matrix [1].

Fibroblast Growth Factors (FGFs) represent a large family of secreted signaling molecules. Its members are classically divided into canonical FGFs (FGF1-10, 16–18, 20 and 22), hormone-like FGFs (FGF15/19,21 and 23) and intracellular FGFs (FGF11-14) [2].

If canonical FGFs signalling *via* high-affinity FGFR receptors (FGFR1-4) exhibit important roles in all cells types, they are best known for the critical role they play in the development of the skeletal system [3]. In contrast, the impact of hormone-like FGFs during skeletogenesis has been hardly investigated. FGF23 has originally been described as a unique FGF subfamily member since it functions as a hormone and required a cofactor, i.e. Klotho, to signal through canonical FGFR [4]. However, a few years later, work from Sitara *et al*. suggested a local role of FGF23 in the skeletal system. Indeed, they demonstrated that *FGF23*^*-/-*^ mice display both a decreased in the number of hypertrophic chondrocytes and a mineralization defect in their growth plate in a phosphate-independent manner [5,6]. Together with the expression of *FGF23* recently reported in hypertrophic chondrocytes [7], this was highly suggestive of a local role of FGF23 in endochondral ossification.

In this work, we made use of ATDC5, a classical *in vitro* model of endochondral differentiation [8], to investigate the expression and role of FGF23 during chondrocyte differentiation. We found that FGF23 was expressed by ATDC5 and had FGFRs-mediated stimulatory effects on ATDC5 differentiation. Our results point out FGF23 as an inducer of hypertrophy and mineralization during chondrogenesis.

## Materials and methods

### Cell culture experiments

The ATDC5 cell line was obtained from ATCC. The cells were maintained in growth medium, named TS medium, made of a 1:1 mixture of DMEM (Dulbecco’s Modified Eagle Medium, Gibco, France) and Ham’s F-12 medium containing 1% (vol/vol) antibiotic-antimycotic (Gibco, France), 5% FBS (Gibco, France), 10 μg/mL transferrin (human transferrin, Sigma-Aldrich, France) and 3.10^−8^ M sodium selenite (sodium selenite, Sigma-Aldrich, France). Cells were maintained in a humidified atmosphere of 5% CO2 at 37°C.

To induce differentiation, the cells were cultured in a differentiation medium, named ITS made of TS medium supplemented with 0.1mg/mL insulin (human insulin solution, Sigma-Aldrich, France). After 14 days (D14) of ITS, the DMEM medium was substituted with α-MEM (Gibco, France) until 21 days (D21) completed by 10mM βGP (Sigma-Aldrich, France) for 7 additional days (D28).

For experiments using micromasses, trypsinized ATDC5 cells were resuspended in ITS medium at a concentration of 2.10^7^ cells/mL. Three drops of 10 μL of this cell suspension were placed in a well of a standard 24-well culture plate. After attachment for 3 h at 37°C, 0.5 mL medium was added to each well. When required, after serum deprivation, cells were stimulated by 100 ng/mL of recombinant mouse FGF23 (rFGF23, R&D Systems, UK) for 24h at 7, 14, 21 or 28 days of culture and treated with 0.5 or 1 μM of PD173074 (Sigma-Aldrich, France), a pan-FGFR inhibitor, during the culture. The medium was renewed every 3 days. Each condition was performed in triplicate.

### RNA extraction and reverse transcription-polymerase chain reaction analysis

Total RNAs were isolated from the ATDC5 cells using RNeasy plus mini kit® (Qiagen, Germany), which allows the total removal of genomic DNA with an on-column DNase. 500ng of total RNA were reverse-transcribed for 90min at 37°C in a 20μl reaction mixture containing 5mM dNTP, 0.2μg/μL random hexamer primers, 250mM Tris-HCl-pH 8.3, KCL 375mM, MgCl2 15mM and 200 units of Moloney Murine Leukemia Virus reverse transcriptase (Invitrogen, USA).

### Real-time quantitative polymerase chain reaction

Real time PCR was performed by the Step One Plus (Applied Biosystems, France) technology using specific primers (Table 1) and iTAQ SYBRgreen master mix system (Biorad, France). All reagents used for RT-PCR were added at the concentrations recommended by the manufacturer (primer concentration was 500 nM each). Melting curve was performed to determine the melting temperature of the specific PCR products and, after amplification (maximum of 40 cycles); the product size was checked on a 1% agarose gel stained with Gel Red (Biotium, Interchim, France). The mRNA levels of the gene of interest and of the *Ribosomal Protein 29* (*RPS29*), chosen as housekeeping gene [9], were determined in parallel for each sample. Quantification was determined using the ΔΔCt method and the results were expressed as fold expression over the control.

**Table 1:** Primer sequences for RT-qPCR.

Oligo sets		Sequences 5’-3’
*RPS29*	For	5’- GGAGTCACCCACGGAAGTT -3’
	Rev	5’- GCCTATGTCCTTCGCGTACT -3’
*COLIIA1*	For	5’- TGG-TAT-TCC-TGG-AGC-CAA-AG-3’
	Rev	5’- ACC-AGT-TGC-ACC-TTG-AGG-AC-3’
*COLXA1*	For	5’- TTC-ATC-CCA-TAC-GCC-ATA-AAG -3’
	Rev	5’- AGG-GAC-CTG-GGT-GTC-CTC -3’
*FGF23*	For	5’-ACC-TGC-CTT-AGA-CTC-CTG-GT-3’
	Rev	5’- GTA-CAG-GTG-GGT-CAG-GCT-TC-3’
*MMP13*	For	5’- ACT-CAA-ATG-GTC-CCA-AAC-GA-3’
	Rev	5’- GGT-GTC-ACA-TCA-GAC-CAG-ACC-3’
*FGFR1*	For	5’- CAC-GAC-CAA-GAA-GCC-AGA-CT-3’
	Rev	5’- CTC-GGC-CGA-AAC-TGT-TAC-CT-3’
*FGFR3*	For	5’- GCA-TCC-TCA-CTG-TGA-CAT-CAA-C-3’
	Rev	5’- CCT-GGC-GAG-TAC-TGC-TCA-AA-3’
*FGFR4*	For	5’- CTC-ACG-GGC-CTT-GTG-AAT-CT-3’
	Rev	5’- CAC-GAA-CCA-CTT-GCC-CAA-AG-3’
*Klotho*	For	5’—AGT-AGA-CGG-GGT-TGT-AGC-CA—3’
	Rev	5’—GGT-TAT-CTG-AGG-CCG-GAT-GG—3’

The 5’-3’ forward and reverse oligonucleotide sequences used for RT-qPCR are listed in Table 1.

### Western blot analysis

After extraction using RIPA buffer, 10 μg of total proteins were separated by 8–12% SDS PAGE and then transferred onto nitrocellulose membrane. After 1h in blocking buffer (5% skim milk in TBS-Tween (TBST)), membranes were washed three times with TBST and incubated overnight at 4°C with primary antibodies against mouse FGFR1, FGFR3, FGFR4 (Abbiotec, USA) used at a dilution of 1/200 or against β-actin (Sigma-Aldrich, France) used at 1/8000. After three 5min-washing steps with TBST, blot were incubated for 1h at room temperature with anti-rabbit IgG conjugated with horseradish peroxidase (Cell Signaling Technology, USA) at 1/4000 dilution in blocking buffer. After three 5min-washes in TBST, signal was detected by chemiluminescence (ECL Plus Western blotting detection reagent, Biorad, France).

### Immunoprecipitation

Immunoprecipitations of FGFRs were performed 14 or 28 days post insulin. Total proteins were extracted using Cell Lysis Buffer (Cell Signalling Technology, USA) containing 20mM Tris (pH 7.5), 150mM NaCl, 1mM EDTA, 1mM EGTA, 1% Triton X-100, 2.5mM Sodium pyrophosphatase, 1mM β-glycerophosphate, 1mM Na3Vo4, 1μg/mL leupeptin and 1mM phenylmethylsulfonyl fluoride (PMSF, Cell Signaling Technology, USA).

Lysates were incubated overnight at 4°C with Phospho-Tyrosine Mouse mAb (magnetic bead conjugate) (P-Tyr-100) (Cell Signaling Technology, USA). Immune-complexes were eluted by Magnetic separation rack and finally eluted off the beads and resolved by SDS-PAGE.

Resolved immunoprecipitates were subjected to Western blotting with primary antibodies directed against FGFR1, FGFR3, FGFR4 (Abbiotec, USA) used at 1/200, ERK½ (Cell Signaling Technology, USA) at 1/500 as non-tyrosine-phosphorylated protein control and secondary anti-rabbit IgG conjugated with horseradish peroxidase (Cell Signaling Technology, USA) at 1/4000. Signal was detected by chemiluminescence (ECL Plus Western blotting detection reagent, Biorad).

### Evaluation of FGF23 production

FGF23 was evaluated in culture media using a mouse Immunoassay (Mouse Fibroblast Growth Factor-23, EZMFGF23-43K Millipore, France). According to manufacturer’s instructions, the sensitivity of the assay in Tissue Culture/Serum/Plasma was 0.69 pg/ml with a range of 137 pg/ml to 100 ng/ml. Our intra-assay precision was 10% and the antibody used in this assay was specific to native mouse FGF23. Supernatant were collected at D0, 7, 14, 21 and 28 and stored at -80°C.

### Alizarin red and alcian blue stainings

ATDC5 cells were plated in 12-well dishes and cultured in ITS differentiation medium for 14 or 28 days. Cells were rinsed with phosphate-buffered saline, fixed 30min in 4% paraformaldehyde and stained with an Alizarin Red solution (1% Alizarin Red S in water, pH 4.2, Sigma, France) for 45min or Alcian Blue solution (0.1% Alcian Blue in 0.1M HCl, pH 1, Sigma, France) overnight.

Quantification of Alizarin Red was obtained at 425nm after coloration dissolution in 10% acetic acid for 30 min, mechanic lysis and then 10% hydroxid ammonium. To quantify Alcian Blue, staining was dissolved in 4M HCl, absorbance was read by Varioskan at 600nm (Thermo Scientific, USA).

Micromasses were fixed in 95% ice-cold methanol for 30 min at 4°C. After washing with water, the micromasses were stained for 1 h in Alizarin red (1% Alizarin Red S in water pH 4.2, Sigma, France). To remove unbound staining, cells were washed with water until the washing solution remained colorless.

### Statistical analyses

Results are expressed as the mean ± SD. Statistical analyses were performed with GraphPad Prism 6 (GraphPad Software) using one-way ANOVA multiple comparisons followed by Tukey correction, or t-test followed by Welch correction. P values were indicated in the legends if considered significant (*<0,05, ** <0,01, *** <0,001).

## Results

### Expression of FGF23 during ATDC5 differentiation

**Fig 1:**
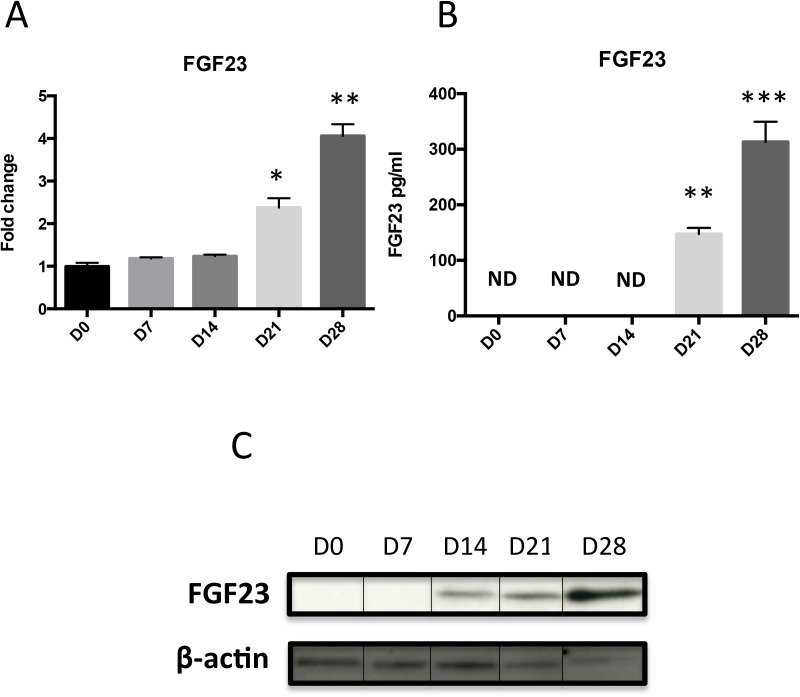
FGF23 expression and production in ATDC5 differentiation. Total RNA was extracted from ATDC5 cultured in ITS conditions for 0, 7, 14, 21 and 28 days, then reverse transcribed into cDNA and analysed by real-time PCR. The relative abundance of FGF23 (A) was normalized to RPS29 mRNA. Comparison was made by using the ΔΔCt method with the fold value of reference (fold = 1) assigned to D0. *: p < 0.05 vs D0, **: p< 0,01 vs D0. Data are expressed as mean ± SD, n = 4. FGF23 production in media was quantified by ELISA (B), **: p< 0,01 vs D0, ***: p< 0,001, ND: Non Detectable. Total proteins were extracted from ATDC5 (0, 7, 14, 21 or 28 days post-insulin) and 10 μg was subjected to SDS-PAGE with FGF23 (1/200) antibody; ß-actin was used as loading control (C).

**Fig 2:**
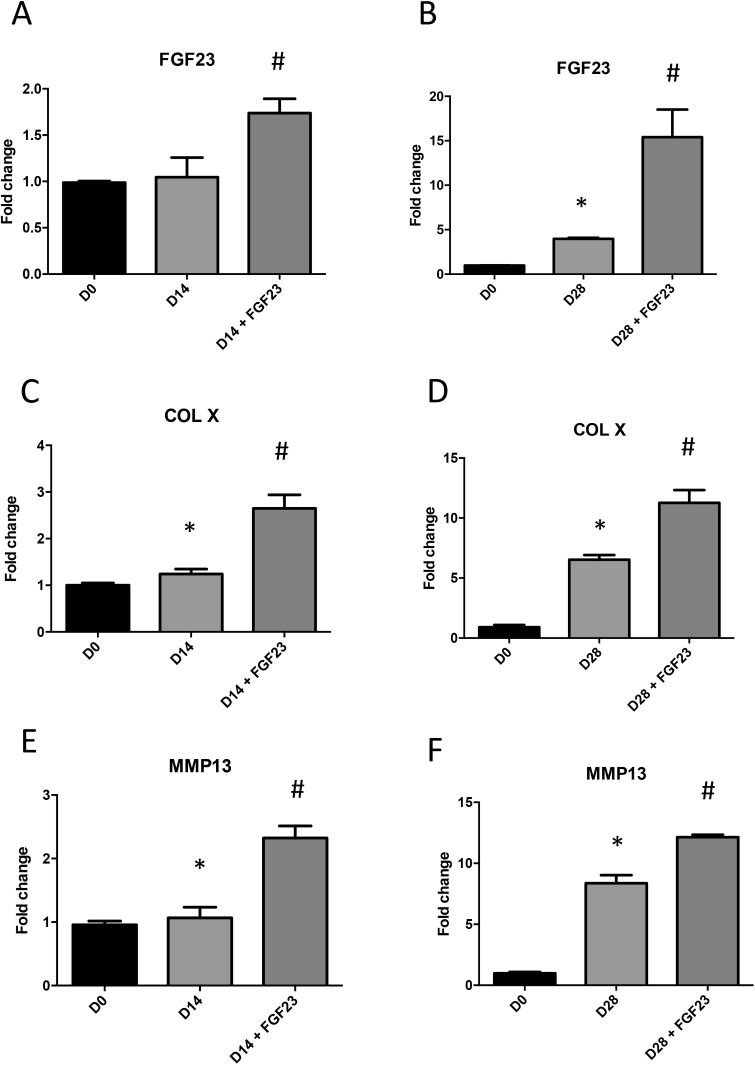
Influence of FGF23 on chondrogenic differentiation. Total RNA was extracted from ATDC5 cultured in ITS conditions for 14 or 28 days and stimulated or not with 100ng/mL mouse rFGF23 for 24h, then reverse transcribed into cDNA and analysed by real-time PCR. The relative abundance at D14 and D28 of FGF23 (A,B), COLX (C,D) and MMP13 (E,F) was normalized to RPS29 mRNA. Comparison was made by using the ΔΔCt method with the fold value of reference (fold = 1) assigned to D0. *: p < 0.05 vs D0, # p<0.05 vs ITS. Data are expressed as mean ± SD, n = 3.

To address the role of FGF23, we first examined its expression over the course of ATDC5 chondrogenic differentiation. When those cells are cultured in ITS conditions, an increase in GAG production and in mineralisation is observed (A in S1 Fig). QPCR analysis showed an increase in *Col II* mRNA at D14, D21 and D28 (B in S1 Fig) and that of specific markers of hypertrophy, namely *Col X* and *MMP13* at D21 and D28 (C and D in S1 Fig). In these very conditions, as summarized on Fig 1, FGF23 expression both at the mRNA and protein levels, increased in a time-dependent manner. Indeed, *FGF23* mRNA increased 2.5 fold at D21 and 4 fold at D28 (Fig 1A, Fig 2A and 2B). ELISA and WB analyses revealed a significant increase of FGF23 from D14-D21 onwards (Fig 1B and 1C).

### Influence of FGF23 on chondrogenic differentiation

We next examined the effect of exogenous FGF23 on the differentiation of ATDC5 by treating the cells with 100 ng/mL mouse rFGF23 for 14 or 28 days in ITS conditions. After rFGF23 stimulation, mRNA coding for *FGF23* was increased 0.5 fold at D14 and 10 fold at D28 compared to ITS medium (Fig 2A and 2B).

Interestingly, expressions of *FGFR1* and *FGFR3* were increased by rFGF23 stimulation in ITS condition at D28 but not at D14 (A-D in S2 Fig), whereas the *FGFR4* expression did not significantly change over time under rFGF23 stimulation (E-F in S2 Fig).

Moreover, the increase of specific markers of hypertrophy, *COL X and MMP13*, observed at D14 (Fig 2C and 2E) and D28 (Fig 2D and 2F) in ITS condition was significantly amplified after stimulation with mouse rFGF23.

### Effect of FGF23 on mineralization

**Fig 3:**
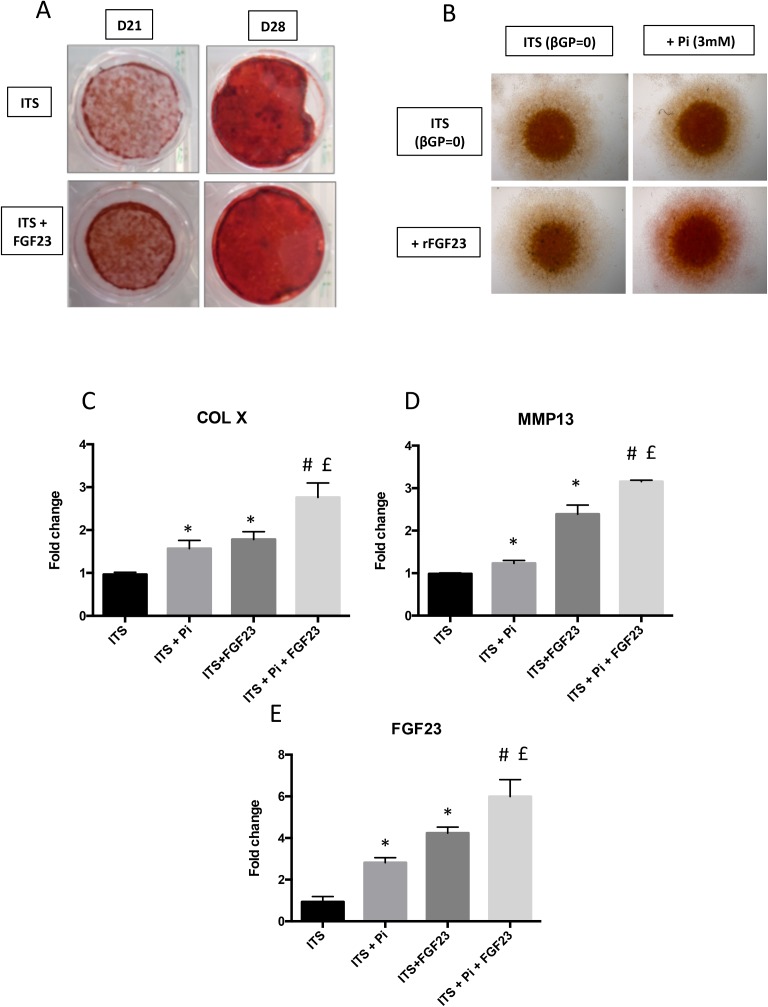
Influence of FGF23 on Pi-induced mineralisation process. ATDC5 were fixed with 4% PFA, and then stained by Alizarin red for 45min after 21 or 28 days of insulin stimulation with or without 100 ng/mL of mouse rFGF23. Images presented are representative of 5 independent experiments (A). ATDC5 micromasses were cultured for 14 days with insulin stimulation in the presence of 3 mM Pi or 100 ng/mL rFGF23 or both. Then they were fixed with 4% PFA and stained by Alizarin red for 45min. Images presented are representative of 3 independent experiments (B). Total RNA was extracted from ATDC5 cultured in ITS conditions for 14 days, then reverse transcribed into cDNA and analysed by real-time PCR and compared to ITS. The relative abundance of COL X, MMP13 and FGF23 were normalized to RPS29 mRNA (C, D, E). Comparison was made by using the ΔΔCt method with the fold value of reference (fold = 1) assigned to ITS. *: p < 0.05 vs ITS, #: p < 0.05 vs ITS + Pi and £: p < 0.05 vs ITS + rFGF23. Data are expressed as mean ± SD, n = 3.

Next, we investigated if FGF23 impacts the very terminal step of ATDC5 differentiation, i.e. mineralization. First, we observed a stronger Alizarin Red staining after stimulation with rFGF23 in ITS condition from D21 (Fig 3A). In ATDC5 micromass experiments, Alizarin Red staining (Fig 3B) showed that co-stimulation with both 100 ng/mL of mouse rFGF23 and 3 mM of Pi, a strong inducer of mineralization [10], induces more pronounced signal than that obtained when those molecules are added independently. If specific markers of the hypertrophic switch, *COL X* and *MMP13*, were induced by Pi and by rFGF23 alone (Fig 3C and 3D), a synergic effect between Pi and rFGF23 stimulation was observed (Fig 3C and 3D) on these markers, substantiating the qualitative data obtained by Alizarin Red staining (Fig 3B). In the same conditions, expression of *FGF23* was increased 3 fold under Pi stimulation alone and 4 fold under rFGF23 stimulation alone (Fig 3E), whereas co-stimulation with both Pi and rFGF23 led to a 6-fold increase of *FGF23* mRNA expression.

### Expression and activation of FGFRs during ATDC5 differentiation

**Fig 4:**
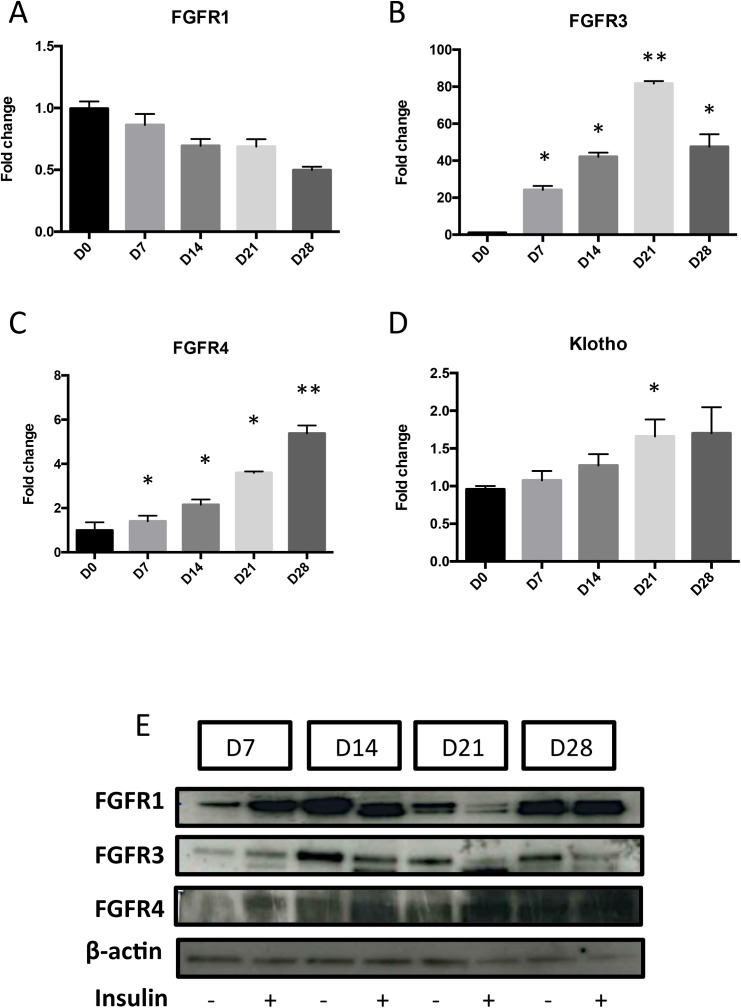
Expression of FGFRs during ATDC5 differentiation. Total RNA was extracted from ATDC5 cultured in ITS conditions for 0, 7, 14, 21 and 28 days, then reverse transcribed into cDNA and analysed by real-time PCR. The relative abundance of FGFR1 (A), FGFR3 (B), FGFR4 (C) and Klotho (D) were normalized to RPS29 mRNA. Comparison was made by using the ΔΔCt method with the fold value of reference (fold = 1) assigned to D0. *: p < 0.05 vs D0, **: p< 0,01 vs D0. Data are expressed as mean ± SD, n = 4. Total proteins were extracted from ATDC5 (7, 14, 21 or 28 days post-insulin) and 10 μg was subjected to SDS-PAGE with anti-FGFR1, FGFR3 or FGFR4 (1/200) antibodies; ß-actin was used as loading control (E).

*FGFR1* (Fig 4A) expression did not display any major modification over 28 days of culture in ITS condition. In contrast, FGFR3 and FGFR4 expressions were increased over time, with a peak at 80 fold (D21) and 5 fold (D28) respectively (Fig 4B and 4C). Western blot using specific antibodies against every FGFR revealed an increase in FGFR1, FGFR3 and FGFR4 expression (Fig 4E). Noteworthy, Klotho (Fig 4D), which is expressed at very low level, is not modulated during differentiation.

**Fig 5:**
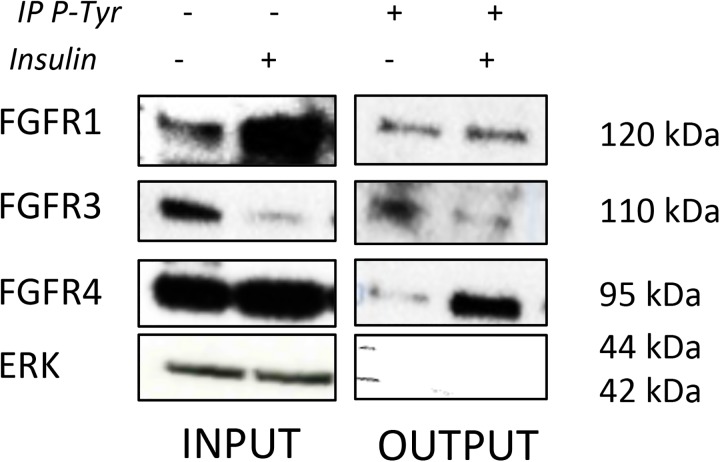
Influence of chondrogenic differentiation on FGFR activation. ATDC5 were cultured for 14 days with insulin. Total protein was then extracted from ATDC5 and subjected to Phospho-Tyrosine Mouse mAb (OUTPUT) or not (INPUT). Antibodies complexes were eluted and resolved by SDS-PAGE with FGFR1, FGFR3, FGFR4 (1/200) or Erk (1/500) antibodies. Erk protein was used as non-tyrosine-phosphorylated protein control.

As the expressions of FGF23 and FGFRs were modified during differentiation of ATDC5, the activated status of each FGFR was investigated by immuno-precipitation (IP) at D14, the earliest time point showing an increase in FGF23 production. IP experiments indicated that activated FGFR4 was strongly increased in ITS-stimulated-cells compared to control at D14. Activated FGFR1 was also increased in control and insulin conditions but no significant differences were observed between these two conditions (Fig 5). When cells were stimulated with rFGF23 at D14, FGFR1 and FGFR4 were strongly activated (S3 Fig).

### FGF23 induces the expression of hypertrophic markers via FGFRs activation

When using 0.5 μM of PD173074, an inhibitor of FGF receptors, during ATDC5 differentiation, mRNA expression of *Col X* was significantly repressed at D14 (Table 2). At D28, a strong inhibitory impact on *MMP13*, *Col X* expression, and on *FGF23* expression and production was observed under FGFRs inhibition (Table 3). Noteworthy, PD173074 impacted FGF receptors activation but not their expression (Tables 2 and 3).

**Table 2:** Effect of specific inhibition of FGFRs activation during ATDC5 differentiation process (Day 14).

PD173074	-	+
	Fold expression	
**Col II**	2.5 ± 0.1	2.3 ± 0.19
**Col X**	2.5 ± 0.35	1.35 ± 0.15 ***
**MMP13**	1.4 ± 0.15	1.32 ± 0.092
**FGFR4**	2.12 ± 0.326	1.9 ± 0.14
**FGF23**	1.2 ± 0.18	1.21 ± 0.11

Total RNA was extracted from ATDC5 cultured in ITS conditions for 14 days treated or not with 0.5 μM PD173074, then reverse transcribed into cDNA and analyzed by real-time PCR. The relative abundance of COL II, COL X, MMP13, FGFR4 and FGF23 was normalized to RPS29 mRNA. Comparison was made by using the ΔΔCt method with the fold value of reference (fold = 1) assigned to D0.

*** p<0,001 vs ITS.

Data are expressed as mean ± SD, n = 3.

**Table 3:** Effect of specific inhibition of FGFRs activation during ATDC5 differentiation process (Day 28).

PD173074	-	+
	Fold expression	
**Col II**	4.2 ± 0.75	3.8 ± 1.8
**Col X**	7.87 ± 1.58	4.2 ± 0.98 ***
**MMP13**	9.7 ± 1.53	4.89 ± 0.86 ***
**FGFR4**	4.1 ± 0.97	4.12 ± 0.98
**FGF23**	4.21 ± 1.59	2.5 ± 0.98*

Total RNA was extracted from ATDC5 cultured in ITS conditions for 28 days treated or not with 0.5 μM PD173074, then reverse transcribed into cDNA and analyzed by real-time PCR. The relative abundance of COL II, COL X, MMP13, FGFR4 and FGF23 was normalized to RPS29 mRNA. Comparison was made by using the ΔΔCt method with the fold value of reference (fold = 1) assigned to D0.

* p<0.05 vs ITS and

*** p<0,001 vs ITS.

Data are expressed as mean ± SD, n = 3.

**Fig 6:**
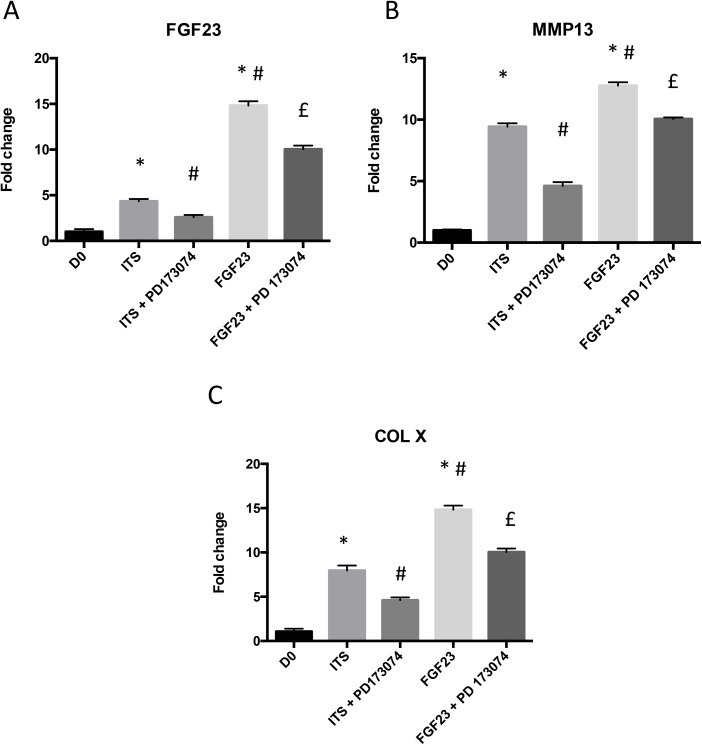
Effect of specific inhibition of FGFR activation during ATDC5 differentiation process and after FGF23 stimulation. Total RNA was extracted from ATDC5 cultured in ITS conditions for 28 days pre-treated or not with 1 μM PD173074 one hour before stimulation with 100 ng/ml of rFGF23 (24h for RNA or 48h for proteins), then reverse transcribed into cDNA and analysed by real-time PCR. The relative abundance of FGF23 (A), MMP13 (B) and COL X (C) was normalized to RPS29 mRNA. Comparison was made by using the ΔΔCt method with the fold value of reference (fold = 1) assigned to D0. *: p < 0.05 vs D0, # p< 0.05 vs ITS, £: p< 0.01 vs rFGF23. Data are expressed as mean ± SD, n = 3.

When cells were pre-treated with 1 μM of PD173074 at D28, rFGF23-induced stimulation of *FGF23* (Fig 6A), *MMP13* (Fig 6B) and *Col X* (Fig 6C) mRNA expression was significantly decreased.

Altogether, these results validate that rFGF23 induces the hypertrophy of ATDC5 cells *via* FGFR activation.

## Discussion

FGF23, the most studied member of the FGF19 subfamily of FGFs, is well known to play a key role in balancing phosphate homeostasis [11]. Besides this systemic effect on phosphate regulation, genetic deletion of *FGF23* have demonstrated that the loss of FGF23 function impaired skeletogenesis [5,6], including a decreased in the number of hypertrophic chondrocytes and mineralization defect of the growth plate. Interestingly, the group of Lanske also elegantly demonstrated that those skeletal defects were phosphate-independent effects, thus implying a local role of FGF23 in endochondral development [5]. More recently, although produced mainly by osteocytes, FGF23 has been shown to be expressed by resting and hypertrophic chondrocytes [7] reinforcing the potential local role for FGF23 on chondrocyte hypertrophy during development. Yet, more data are warranted to dissect the role FGF23 on chondrocyte differentiation. Thus, in order to investigate the potential role of FGF23 during chondrogenesis, we made use of ATDC5 cells, an *in vitro* model that recapitulates all differentiation steps of chondrocytes [8]. Indeed when cultured with insulin, these cells express over time moderate [12] and in our present study, to high level of chondrocyte hypertrophic markers.

In the present study, we show for the first time an increased expression of FGF23 mRNA and protein levels during ATDC5 differentiation. Indeed, FGF23 expression and production was shown to gradually increase during endochondral differentiation from D14 onwards. These results are in agreement with the *in vivo* expression occurring in the growth plate [7]. Then, we observed that stimulation of ATDC5 cells with exogenous rFGF23 induces the expression of the hypertrophic markers, *MMP13* and *Col X*. Those results are in agreement with data that we obtained recently on osteoarthritic chondrocytes [13] and are also consistent with the reciprocal results observed in the growth plate of *FGF23*^*-/-*^ mice, where *Col X* expression is in contrast strongly reduced [5,6].

Following hypertrophy, terminal differentiation is marked by the mineralization of the extracellular matrix. Our results demonstrated that, provided ßGP is present, FGF23 alone is able to positively regulate mineralization of ATDC5. However, we showed that the FGF23-mediated mineralization is increased in the presence of Pi, a known modulator of terminal differentiation including hypertrophy and mineralization [11,14]. Apart from this interesting apparent synergy observed between FGF23 and Pi, this raises the question whether FGF23, that is a pivotal regulator of systemic Pi homeostasis, may also regulates local Pi concentrations in the chondrocyte environment.

To further understand FGF23-induced hypertrophy and mineralization mechanism, we studied FGFRs expression and activation during ATDC5 differentiation. First, we show an increased expression of FGFR1 (at D7, D14 and D28) and FGFR3 (D7-D21) during ATDC5 differentiation. These results are congruous with FGFRs expression observed during *in vivo* endochondral differentiation and similar to those from other previous reports that looked at the expression of FGFRs and their isoforms in ATDC5 [14–16]. In addition, although it has not been reported previously, we were able to detect the expression of FGFR4 during ATDC5 differentiation with a particularly strong activation of this receptor at D14, the time point where FGF23 production initiates. Noteworthy, as shown previously by others [17] and confirmed in the present study, klotho is very faintly expressed in ATDC5. Overall, this suggests that FGF23 signals through FGFR4 in the absence of klotho in ATDC5. Together with our recent report on human chondrocytes [13], that defines chondrocyte as a cell capable of responding to FGF23 signaling in a klotho-independent manner. Indeed, although FGFR1 and Klotho have been known to be obligatory proteins for FGF23 actions [2], our results are in accordance with recent studies reporting that FGFR4 mediates a klotho-independent FGF23 signaling in cardiomyocytes or hepatocytes [18–20].

To confirm the role of FGF23 in promoting terminal differentiation of ATDC5, we analyzed the modulation of its effects in presence of an FGFR inhibitor, namely PD173074. The addition of PD173074, in both conditions where FGF23 was endogenously produced (ITS) or exogenously provided (rFGF23), was able to significantly reduced the increased mRNA expression of *Col X* and *MMP13* observed in the absence of the inhibitor. The partial inhibition obtained is likely attributable to the numerous molecules that are non-members of the FGF family, produced by ATDC5 over the course of their differentiation and are also able to induce *Col X* and *MMP13* in these cells.

Further experiments, via shRNA and specific blocking antibodies strategies, are still required to establish the precise contribution of endogenous FGF23 and its FGFRs during ATDC5 differentiation.

In addition to other FGFs such as FGF2, FGF9 and FGF18 previously reported to influence ATDC5 differentiation [14], the present study provides evidence that endogenous FGF23 is produced by ATDC5 in rather late stages chondrogenesis. This expression pattern together with the results of our experiments using exogenous rFGF23 suggest that FGF23 may participate in the terminal differentiation of these cells. Together with our recent report on human osteoarthritic chondrocytes [9], this authenticates FGF23 as a new FGF member implicated in cartilage hypertrophy and mineralization.

## Supporting information

S1 Fig Influence of chondrogenic differentiation on mineralization, GAG production and expression of specific makers. ATDC5 were fixed with 4% PFA, and then stained by Alizarin red for 45min or Alcian Blue overnight after 7, 14, 21 or 28 days of insulin induction. Results presented are representative of 3 independent experiments (A). Quantification of Alizarin Red was obtained by 10% acetic acid for 30 min, mechanic lysis and then 10% hydroxide ammonium. To quantify Alcian Blue, cell lysate was dissolved in 4M HCl. Absorbance was read on Varioskan and results are presented in histograms as means (±SD), (n = 5). Total RNA was extracted from ATDC5 cultured in ITS conditions for 0, 7, 14, 21 and 28 days, then reverse transcribed into cDNA and analysed by real-time PCR and compared to D0. The relative abundance of COL II, COL X and MMP13 was normalized to RPS29 mRNA (B, C, D). Comparison was made by using the ΔΔCt method with the fold value of reference (fold = 1) assigned to D0. *: p < 0.05 vs D0, **: p< 0,01 vs D0. Data are expressed as mean ± SD, n = 5.
(TIF)

S2 Fig Influence of FGF23 on FGFRs expression in ATDC5 differentiation. Total RNA was extracted from ATDC5 cultured in ITS conditions for 14 or 28 days and stimulated or not with 100ng/mL mouse FGF23 for 24h, then reverse transcribed into cDNA and analysed by real-time PCR and compared to D0. The relative abundance of FGFR1 (A,B), FGFR3 (C,D) and FGFR4 (E, F) was normalized to RPS29 mRNA. Comparison was made by using the ΔΔCt method with the fold value of reference (fold = 1) assigned to D0. *: p < 0.05 vs TS, #: p<0.05 vs ITS. Data are expressed as mean ± SD, n = 3.
(TIF)

S3 Fig Influence of FGF23 on FGFRs activation in ATDC5 differentiation. ATDC5 were cultured with insulin for 14 days with or without 100 ng/mL of mouse rFGF23. Total protein was then extracted from ATDC5 and subjected to Phospho-Tyrosine Mouse mAb (OUTPUT) or not (INPUT). Antibodies complexes were eluted and resolved by SDS-PAGE with FGFR1, FGF FGFR4 (1/200) or Erk (1/500) antibodies. Erk protein was used as non-tyrosine-phosphorylated protein control.
(TIF)
