# Supplementary figures and images for: Fibroblast-growth factor 23 promotes terminal differentiation of ATDC5 cells
Source: PLoS One. 2017 Apr 13;12(4):e0174969. doi: 10.1371/journal.pone.0174969 (PMC5390990; doi:10.1371/journal.pone.0174969)

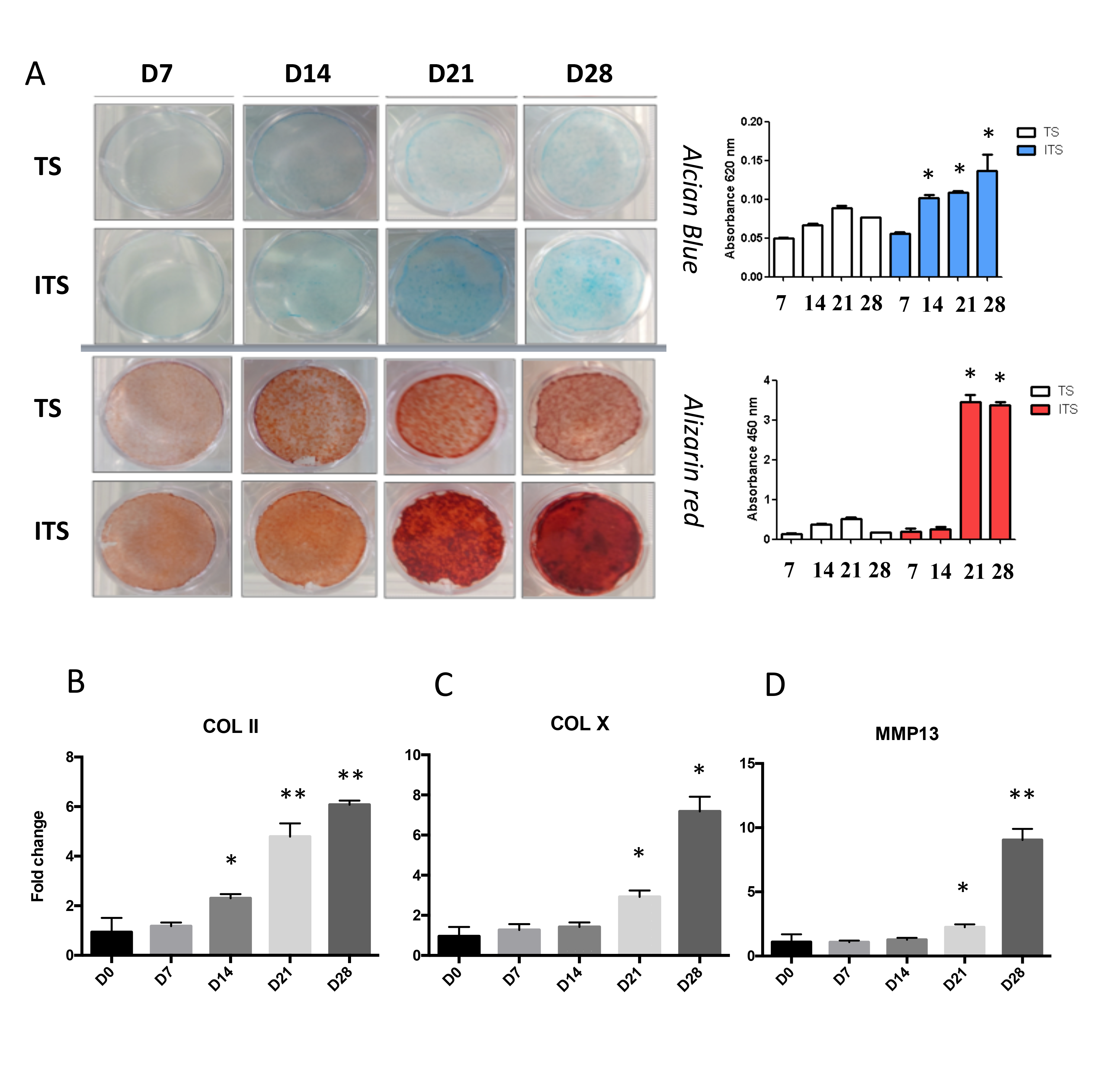

Supplement: S1 Fig — ATDC5 were fixed with 4% PFA, and then stained by Alizarin red for 45min or Alcian Blue overnight after 7, 14, 21 or 28 days of insulin induction. Results presented are representative of 3 independent experiments (A). Quantification of Alizarin Red was obtained by 10% acetic acid for 30 min, mechanic lysis and then 10% hydroxide ammonium. To quantify Alcian Blue, cell lysate was dissolved in 4M HCl. Absorbance was read on Varioskan and results are presented in histograms as means (±SD), (n = 5). Total RNA was extracted from ATDC5 cultured in ITS conditions for 0, 7, 14, 21 and 28 days, then reverse transcribed into cDNA and analysed by real-time PCR and compared to D0. The relative abundance of COL II, COL X and MMP13 was normalized to RPS29 mRNA (B, C, D). Comparison was made by using the ΔΔCt method with the fold value of reference (fold = 1) assigned to D0. *: p < 0.05 vs D0, **: p< 0,01 vs D0. Data are expressed as mean ± SD, n = 5. (TIF) [file pone.0174969.s001.tif]

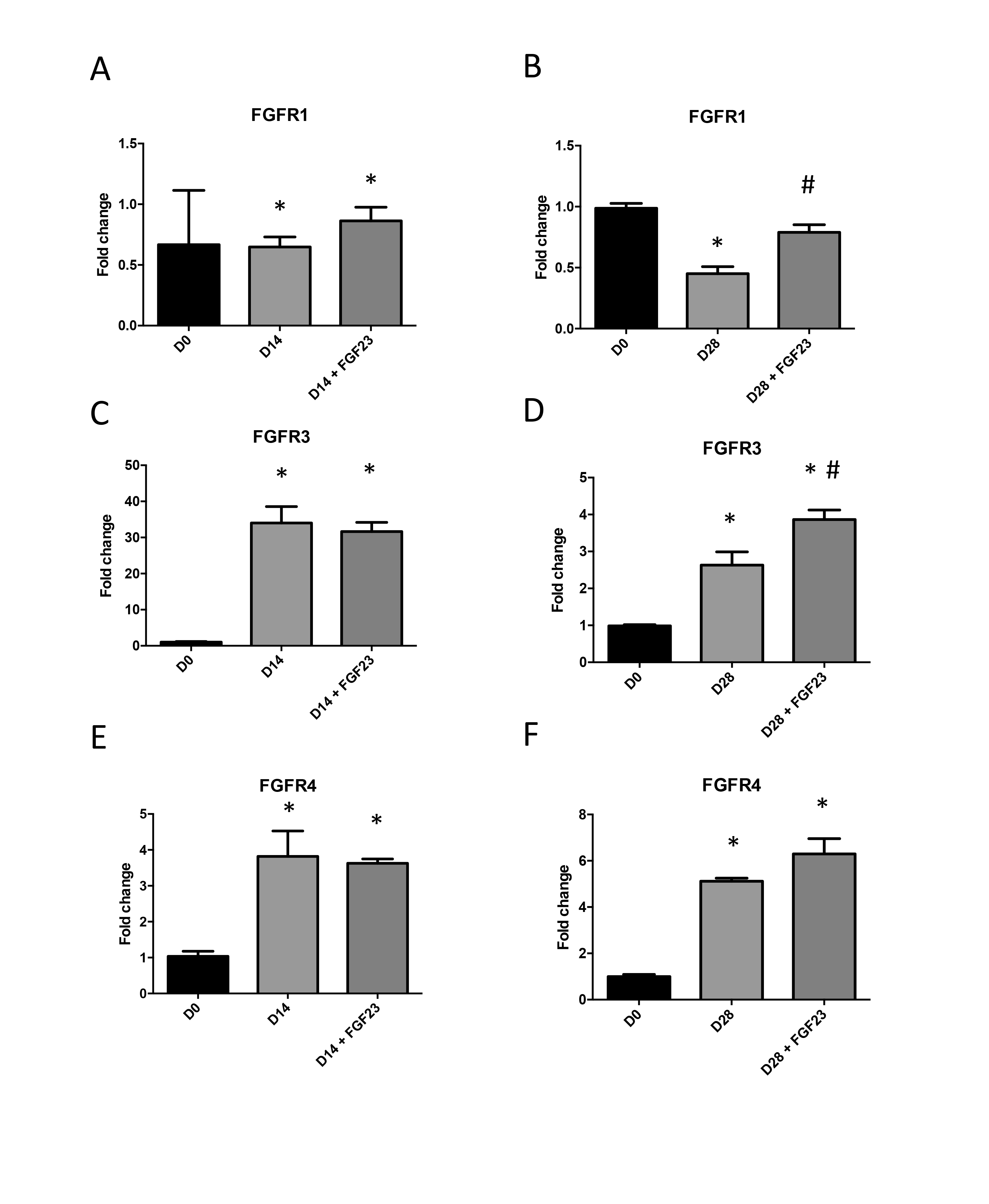

Supplement: S2 Fig — Total RNA was extracted from ATDC5 cultured in ITS conditions for 14 or 28 days and stimulated or not with 100ng/mL mouse FGF23 for 24h, then reverse transcribed into cDNA and analysed by real-time PCR and compared to D0. The relative abundance of FGFR1 (A,B), FGFR3 (C,D) and FGFR4 (E, F) was normalized to RPS29 mRNA. Comparison was made by using the ΔΔCt method with the fold value of reference (fold = 1) assigned to D0. *: p < 0.05 vs TS, #: p<0.05 vs ITS. Data are expressed as mean ± SD, n = 3. (TIF) [file pone.0174969.s002.tif]

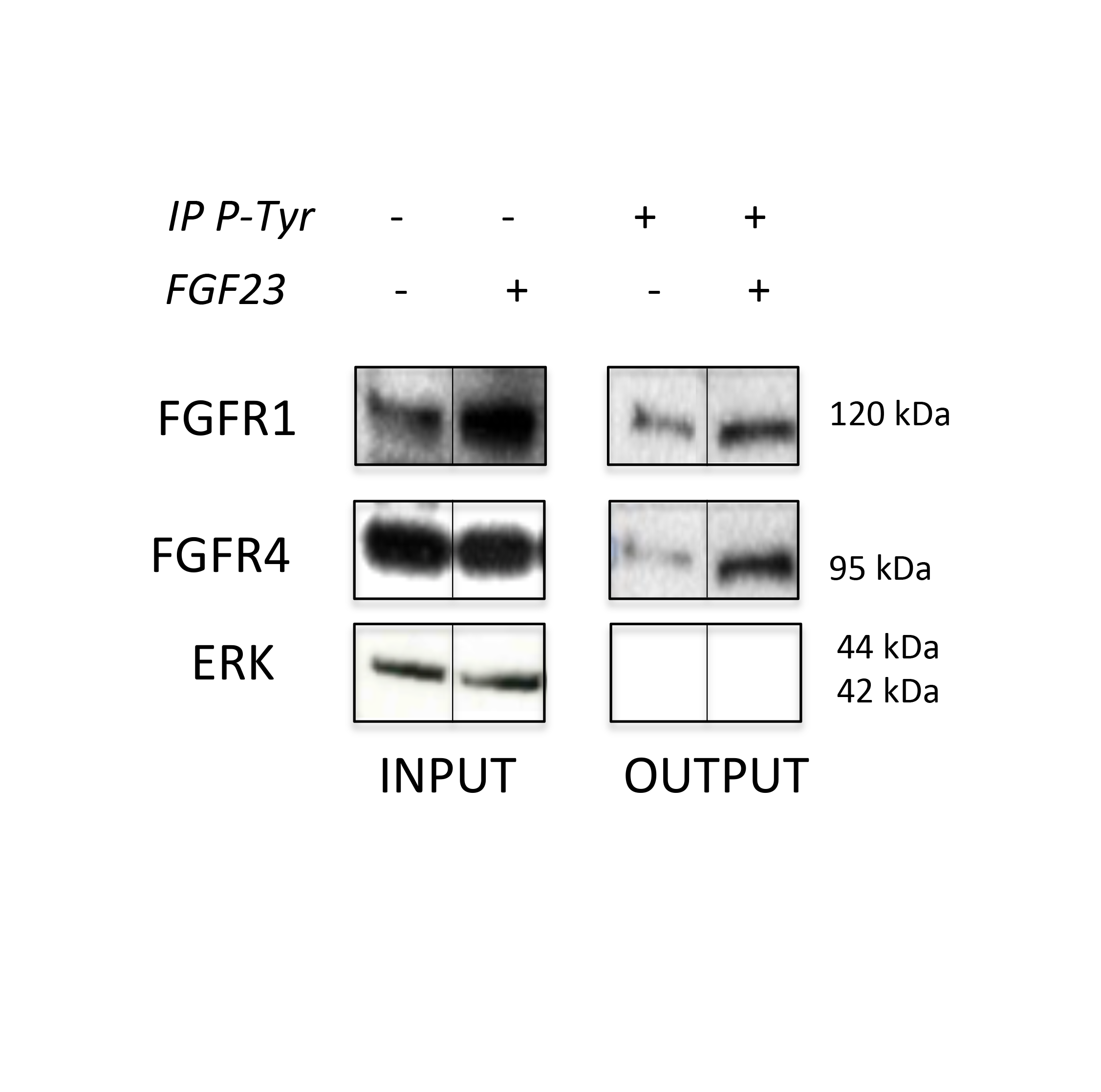

Supplement: S3 Fig — ATDC5 were cultured with insulin for 14 days with or without 100 ng/mL of mouse rFGF23. Total protein was then extracted from ATDC5 and subjected to Phospho-Tyrosine Mouse mAb (OUTPUT) or not (INPUT). Antibodies complexes were eluted and resolved by SDS-PAGE with FGFR1, FGF FGFR4 (1/200) or Erk (1/500) antibodies. Erk protein was used as non-tyrosine-phosphorylated protein control. (TIF) [file pone.0174969.s003.tif]
